# Admissions to a Low-Resource Neonatal Unit in Malawi Using a Mobile App: Digital Perinatal Outcome Audit

**DOI:** 10.2196/16485

**Published:** 2020-10-21

**Authors:** Caroline Crehan, Erin Kesler, Indira Angela Chikomoni, Kristi Sun, Queen Dube, Monica Lakhanpaul, Michelle Heys

**Affiliations:** 1 UCL-Great Ormond Street Hospital Institute of Child Health University College London London United Kingdom; 2 Zomba Central Hospital Zomba Malawi; 3 Whittington Hospital London United Kingdom; 4 Paediatric Department College of Medicine University of Malawi Blantyre Malawi; 5 East London NHS Foundation Trust London United Kingdom

**Keywords:** infant, newborn, mHealth, data collection, clinical audit, digital health, low income population, mobile phone

## Abstract

**Background:**

Mobile health (mHealth) is showing increasing potential to address health outcomes in underresourced settings as smartphone coverage increases. The NeoTree is an mHealth app codeveloped in Malawi to improve the quality of newborn care at the point of admission to neonatal units. When collecting vital demographic and clinical data, this interactive platform provides clinical decision support and training for the end users (health care professionals [HCPs]), according to evidence-based national and international guidelines.

**Objective:**

This study aims to examine 1 month’s data collected using NeoTree in an outcome audit of babies admitted to a district-level neonatal nursery in Malawi and to demonstrate proof of concept of digital outcome audit data in this setting.

**Methods:**

Using a phased approach over 1 month (November 21-December 19, 2016), frontline HCPs were trained and supported to use NeoTree to admit newborns. Discharge data were collected by the research team using a discharge form within NeoTree, called *NeoDischarge*. We conducted a descriptive analysis of the exported pseudoanonymized data and presented it to the newborn care department as a digital outcome audit.

**Results:**

Of 191 total admissions, 134 (70.2%) admissions were completed using NeoTree, and 129 (67.5%) were exported and analyzed. Of 121 patients for whom outcome data were available, 102 (84.3%) were discharged alive. The overall case fatality rate was 93 per 1000 admitted babies. Prematurity with respiratory distress syndrome, birth asphyxia, and neonatal sepsis contributed to 25% (3/12), 58% (7/12), and 8% (1/12) of deaths, respectively. Data were more than 90% complete for all fields. Deaths may have been underreported because of phased implementation and some families of babies with imminent deaths self-discharging home. Detailed characterization of the data enabled departmental discussion of modifiable factors for quality improvement, for example, improved thermoregulation of infants.

**Conclusions:**

This digital outcome audit demonstrates that data can be captured digitally at the bedside by HCPs in underresourced newborn facilities, and these data can contribute to a meaningful review of the quality of care, outcomes, and potential modifiable factors. Coverage may be improved during future implementation by streamlining the admission process to be solely via digital format. Our results present a new methodology for newborn audits in low-resource settings and are a proof of concept for a novel newborn data system in these settings.

## Introduction

### Background

Each year, 2.5 million newborns die with no registration of their death or any documentation of how or why they died [1]. Half of the world’s newborn babies do not receive a birth certificate [2,3], and stillbirths are statistically invisible [3]. Despite this, it is widely acknowledged that to prevent newborn deaths, more information on the number of births and deaths, causes of deaths, and avoidable factors linked to deaths is needed [4]. The process of a perinatal death audit aims to establish the profile of facility-based causes of death and has been shown to reduce perinatal mortality by 30% in low-resource countries [5]. Improving the quality of care for newborns is a key priority in tackling persistently high neonatal mortality rates (NMRs), particularly for hospitalized sick newborns in low- and middle-income countries [6]. At the facility level, the process of audit and feedback is considered the cornerstone of quality improvement, particularly when the process includes a clear action plan and targets [4]. Meanwhile, smartphone technology is becoming increasingly commonplace in low-resource settings. Mobile health (mHealth; “the use of mobile and wireless technologies to support the achievement of health objectives” [7]) has been harnessed for accurate and efficient data collection, particularly in community settings and in the context of clinical trials [8,9]. To the best of our knowledge, mHealth has not been utilized in the context of a hospital perinatal death audit. In response to recognized challenges in the scale-up of audits in these settings and a call for electronic health systems [5], we present the results of a novel digital outcome audit collected by health care professionals (HCPs) on an mHealth app: the NeoTree [10].

**Figure 1:**
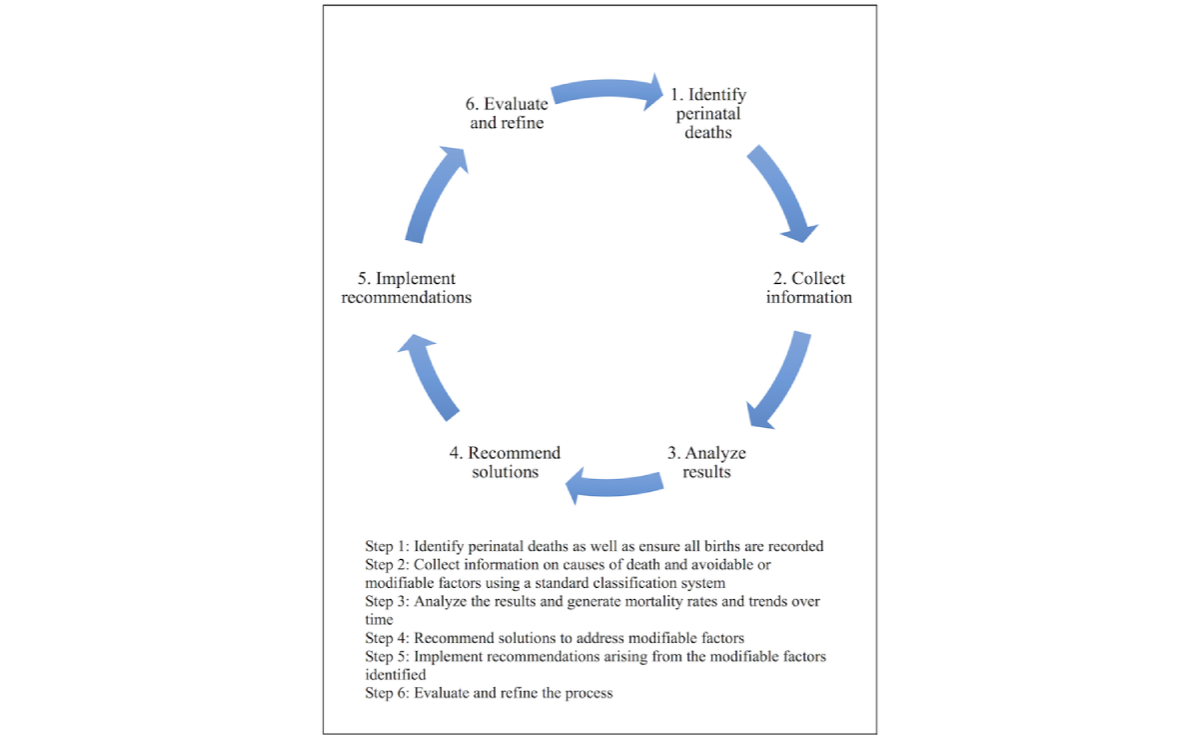
Six-step cycle for perinatal mortality audits.

Different types of clinical audits exist for different purposes, for example, a structural audit examines the availability of resources in a system and a process audit assesses the process of case management [5]. An outcome audit assesses the end results of care, either deaths or near misses, depending on the NMR. In high-income countries where the NMR is low, the focus is more on near misses, and the management of specific cases are typically discussed in a monthly multidisciplinary *morbidity and mortality* *meeting*. In low-resource settings where the NMR is high, the emphasis is on deaths, and these are often discussed in a *death audit* meeting. Perinatal death audits have been defined as “the process of capturing information on the number and causes of all still births and neonatal deaths, or near misses where applicable, with an aim toward identifying specific cases for systematic, critical analysis of the quality of perinatal care received in order to improve the care provided to all mothers and babies” [4]. It aims to follow a 6-step cycle summarized in Figure 1 (adapted from Pattinson et al [5]).

Despite being one of the least developed countries in the world, Malawi has seen great success in achieving the Millennium Development Goal 4 [11], particularly for underfive mortality; however, for newborns, mortality is persistently high. A previous pediatric death audit in Kamuzu Central Hospital (KCH), a large referral center in Malawi, was recently published and cited reliable records as a significant limitation, with 6% of charts missing and documentation deficiencies in 58% of the charts reviewed [12]. To our knowledge, a perinatal death audit has not yet been published from Malawi or other sub-Saharan countries.

### Objectives

Our objective was to conduct and report a digital outcome audit of admission and discharge information collected by HCPs in a district-level facility using a novel mHealth app, NeoTree, on electronic mobile devices.

## Methods

### Setting

Zomba Central Hospital (ZCH) is a district-level hospital in Malawi with a large neonatal unit. During our study period and in discussions with the local clinical management team, it was noted by the researcher-in-residence (CC) that the 40-bed nursery takes around 7 admissions per day with 9 staff covering day and night shifts on 3 neonatal wards (high dependency, transit, and kangaroo wards). At maximum capacity, this equates to up to 3 nurses looking after approximately 50 babies per shift. Admissions are referred from a range of areas, defined as their *place of origin*, from within ZCH (eg, Theater), or from outside ZCH (eg, home, health center, or other hospital). A health center in this context is a facility that provides outpatient care services for common diseases in the local population, whereas a hospital is a larger facility providing more specialized care to a district population. Admissions are usually clerked on a Malawian Ministry of Health (MOH) paper proforma. Each patient’s paper-based medical record consists of this admission form and any other loose-leaf sheets held together with a string. The records of NNDs are examined by pediatric and neonatal HCPs at monthly death audit meetings, often without an obstetric input. A rudimentary root cause analysis for each death was postulated, and subsequent recommendations were made. It was observed informally by the researcher-in-residence (CC) that the quality of the paper records is very poor [12], with faded, illegible writing, and the death audit process is time-consuming and often takes a whole day. She also observed that clinicians find it particularly difficult to attend death audits as their clinical duties continue. Oxygen concentrators and heaters in the unit rely on an electricity supply, which is affected by power outages on a daily basis. When this occurs, a backup supply of electricity is provided by a generator within a few minutes.

### Identifying Deaths and Collecting Information Using a Digital App: Stages 1 and 2 of the Audit Cycle

#### The NeoTree App

A bedside app, NeoTree, was used (in addition to the MOH paper admission form) by frontline neonatal staff (mainly nurses) to record 1 month of admissions (November 21-December 19, 2016) to the neonatal unit on 3 low-cost Android electronic tablet devices, which were provided and installed at the nursing station. This audit took place in the context of an intervention development study [10], which presents the co-development process and acceptability, feasibility, and usability data.

**Figure 2:**
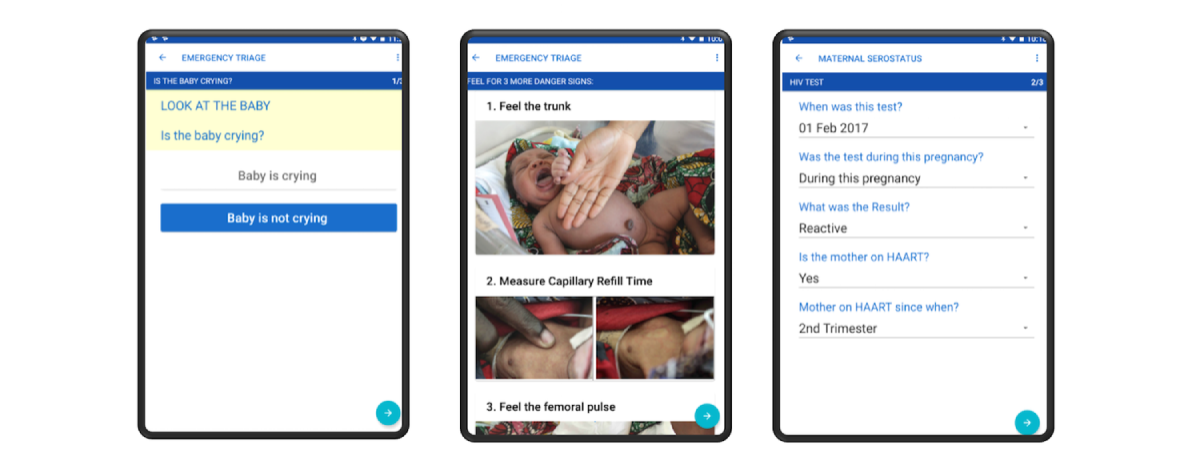
Example app screens.

The NeoTree app was structured as an electronic admission form (Figure 2), which included all the fields of the standardized MOH paper neonatal admission form. Owing to HCP feedback, some fields were removed (eg, physical gestational score), but no new fields were added. By the end of the study period, the digital form followed the structure as below:

- Emergency triage and vital signs.
- Patient identification and demographics.
- Reason for admission (presenting history).
- Examination.
- Place of origin.
- Maternal history.
- Provisional HCP admission diagnoses.

#### Digital Admission Form

Most fields on the electronic admission form were compulsory in that the HCP users could not continue until a plausible value had been recorded. However, in attempts to make the form more user-friendly and practical, some fields that relied on the availability of specific equipment (such as blood sugar and oxygen saturations) were made optional. Data collected at admission and discharge were stored locally on the tablets, and a printed copy (printed via a wireless printer on the ward) was kept in each patient’s paper-based medical record, which included patient identifiers. When a network connection became available, data were exported pseudoanonymized with a unique ID to the researcher-in-residence’s (CC) laptop as a file using Excel (Microsoft) program. Fields containing identifiable information were preconfigured as confidential by the researcher-in-residence so that they were not exported.

During each digital admission, a *reason for admission* was recorded. This was the presenting complaint entered by the nurse according to what the baby was referred to from the labor ward or other facility. If the referred patient arrived without accompanying information, the HCPs had to use their own clinical judgment. The reason for admission may have differed considerably from the actual diagnosis, where, for example, labor ward referrals were labeled *meconium aspiration* simply because there was meconium at delivery. The reason for admission was mutually exclusive, as there was only 1 presenting complaint recorded for each baby. Later, at the end of the digital admission form, *provisional HCP admission diagnoses* were decided by the HCPs based on their assessment of the baby. They could choose more than one diagnosis when necessary; hence, they were not mutually exclusive.

During the admission process, gestation was estimated from fundal height (recorded antenatally in the maternal record) or length of pregnancy (reported by the mother), both of which are unreliable methods [13,14]; hence, a physical maturity score was included in early iterations of NeoTree (Multimedia Appendix 1). Occasionally, wireless printing of forms was momentarily delayed by power outages, but these did not affect the completion of forms on the tablets because they had an 8-hour battery life.

#### Digital Discharge Form

The discharge form, the *NeoDischarge*, was completed by a researcher (EK) upon review of the patient’s record just after discharge or death. Discharge information collected included identifiable information, outcome (discharged alive, absconded, or dead), HCP discharge diagnoses 1, 2, and 3 for discharges, and cause of death for NNDs. Often, there was more than one HCP discharge diagnosis documented; hence, the need for 3 fields, and these data were not mutually exclusive. It was noted that some patients in the sample had not had a documented review by a clinician during admission; hence, the researcher-in-residence (CC, a UK pediatrician in training with 4 years of neonatal experience, including 18 months in low income country newborn care), decided the most salient singular discharge diagnosis for all babies. This was labeled *researcher-in-residence discharge diagnosis*.

### Recruitment and Initial Training

All 9 permanent staff on the neonatal rota were invited to attend a scenario-based one-on-one session in which they were trained to record new admissions using the app. Written consent to participate in the study was obtained according to ethical approval from The Malawi College of Medicine Research and Ethics Committee (reference 17PP12). Each HCP was then supervised clerking (recording) 1 new admission on the ward, completing both the MOH paper form and the form on the NeoTree app. When patients arrived with partially completed paper forms, HCPs were encouraged to cross-check information already documented, with mothers and the mother’s medical record book, before entering it to the app. Permanent staff who could not attend the initial training session gave written consent when they entered the study (n=1). The audit and implementation were explained to other ad hoc locum staff or nursing students by the head nurse of each shift, who obtained their verbal consent.

### Phased Implementation and Iterative Changes

NeoTree was then implemented on the ward in a phased approach over 1 month, with increasing coverage and decreasing levels of supervision. In the first week of the study, day-time admissions were supervised by the researcher-in-residence (CC) who was present in the ward. She was also available throughout the 1-month study to support any technical issues that occurred. Electronically clerked admissions were cross-checked by the researcher-in-residence with the admissions book and the ward manager at the end of each day to see if any patients had not been captured on NeoTree. Incomplete sessions were deleted. During the study, via a web-based editor platform, the researcher-in-residence was able to act on verbal feedback from the nurses as they used the app, and reconfigure NeoTree to best fit an efficient admission process in consultation with the wider NeoTree team. For example, the order of sections was adjusted to start with triage and examination and end with mothers’ history (the opposite of the order of the MOH paper form), and pictures were added to explain exactly how to check danger signs.

### Statistical Analysis: Stage 3 of the Audit Cycle

The primary outcome of this study was to report and describe deaths over a 1-month period using an electronic app, NeoTree. To this end, the admission and outcome datasets were exported from the app and merged by matching unique identification numbers. The SPSS program was then used to analyze the data and produce simple graphs [15]. Descriptive statistics included totals (n), percentages (%), mean and SD for normally distributed data, and median and IQR for skewed data and ranges. Charts included simple bar and pie charts for categorical data and histograms for continuous data (eg, a histogram for temperature). To measure the digital process, the number of missing pieces of data for each data point was reported as a total (n) and as a percentage (%). The average time taken to complete the app was also calculated. Case fatality rates (CFRs) for each diagnosis were calculated as the number of deaths per 1000 babies admitted with that diagnosis, for example, CFR for respiratory distress syndrome (RDS) was 148 deaths per 1000 cases of RDS. The overall CFR was calculated as the number of deaths per 1000 babies admitted.

### Presentation of Results at an Audit Meeting: Stage 4 of the Audit Cycle

Simple graphs and statistics produced were presented by the researcher-in-residence (CC) to the department 2 weeks later in conjunction with that month’s death audit meeting. The researcher facilitated the discussion regarding each graph sequentially; however, the discussion was led by the participants. Emerging patterns in admission and outcome data, CFRs, and modifiable factors contributing to morbidity and mortality were identified, and possible solutions were discussed. Individual cases were not discussed.

## Results

### Participants

A total of 31 HCPs from 4 different cadres clerked newborns using the NeoTree app, including all 9 permanent staff on the rota, 8 of whom attended the one-on-one training workshops at the beginning of the study. All the other 22 participants were nonpermanent locum staff or nursing students temporarily working on the unit (Table 1).

Participants attending the audit meeting included 3 female nurse midwife technicians, 1 male clinical officer (who was also the head of the department), and 1 male nursing officer.

**Table 1:** Participant characteristics.

Characteristics		Values (N=31)
Gender (female), n (%)		22 (73)
Age (years), mean (SD); range		30 (12.3); 19-65
**Cadre, n (%)**		
	Nurse midwife technician	7 (23)
	Nursing officer	6 (19)
	Nurse	1 (3)
	Student-nurse	17 (55)
Years of neonatal experience, mean (SD); range		1 (4.4); 0-8
Previously used a tablet device, n (%)		15 (50)
Regularly used a tablet device, n (%)		4 (13)
Received COIN^a^ training, n (%)		5 (16)
Received HBB^b^ training, n (%)		22 (71)

^a^COIN: care of the infant newborn [16].

^b^HBB: helping babies breathe.

### Digital Outcome Audit Findings: Stage 3 of the Audit Cycle

During the study period, there were 191 admissions to the neonatal ward. A total of 70.2% (134/191) admissions were completed using NeoTree, and data from 130 babies were exported for analysis. Of these, 129 were analyzed because of 1 repeat entry. Hence, 67.5% (129/191) of all admissions were analyzed.

Table 2 describes the patient demographics and clinical data. The key highlights are described here. The mean birth weight was 2616 g, with just over one-third 37.6% (44/117) of babies born at low birth weight (LBW) and 45.0% (58/129) born prematurely at <37 weeks’ gestation. In total, 25 maturity scores were carried out by HCPs, most of which estimated higher gestation than the fundal height or length of pregnancy methods (data not shown).

**Figure 3:**
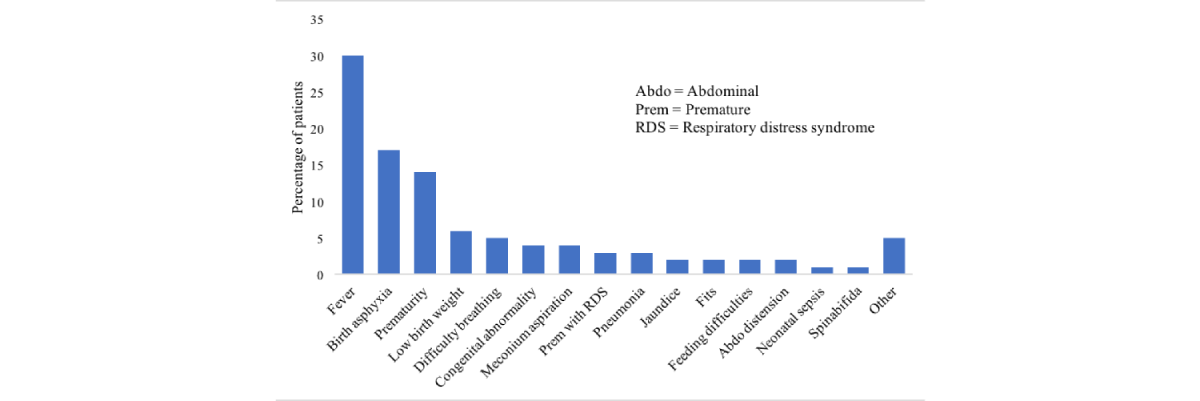
Reasons for admission to neonatal ward.

**Figure 4:**
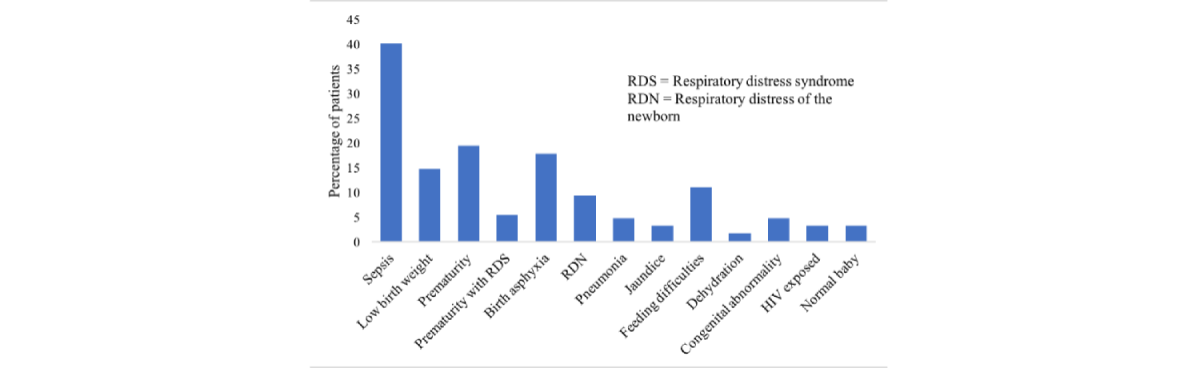
Provisional health care professional admission diagnoses (not mutually exclusive).

Figure 3 depicts the clinical reason for admission, recorded at the beginning of the app before the clinical assessment, the most common of which was fever 30.2% (39/129), followed by birth asphyxia 17.1% (22/129) and prematurity 14.0% (18/129). The provisional admission diagnoses made by HCPs (at the end of the app, after clinical assessment) are reported in Figure 4.

The most common discharge diagnoses recorded by the HCP and the researcher-in-residence were very similar (Table 2): sepsis followed by birth asphyxia and LBW. In total, 84.3% (102/121) babies were discharged alive, 5.8% (7/121) left the hospital against medical advice, and 9.9% (12/121) died.

Table 3 describes the clinical findings at the emergency triage and HIV status of the babies. Two-third of the babies did not cry at triage and hence underwent an airway, breathing, circulation, and disability examination with only a minority considered *unstable* following this assessment. Grunting was the most common danger sign in unstable babies. Over a third (49/129, 38.0%) of neonates admitted were hypothermic. A fifth of the babies were known to be exposed to HIV before or during birth.

The cause of death for the 12 newborn deaths, as recorded by the second researcher (EK) on the discharge form, are shown in Table 4. In all 12 patients, the cause of death was the same as the researcher-in-residence’s discharge diagnosis. Prematurity with RDS and birth asphyxia were the leading causes of death.

**Table 2:** Patient demographics and main findings of neonatal admissions.

Characteristics			Findings, n (%)		Missing (n=129), n (%)		Complete (n=129), n (%)
**Demographics (n=129)**							
	**Gender, n (%)**			0 (0)		129 (100)	
		Male	79 (61.2)				
		Female	50 (38.7)				
	**Age (hours), n (%)**			0 (0)		129 (100)	
		≤48	85 (65.9)				
		>48	44 (34.1)				
	**Type of birth, n (%)**			0 (0)		129 (100)	
		Singletons	124 (96.1)				
		Twins	5 (3.8)				
	**Admitted from, n (%)**			0 (0)		129 (100)	
		Within Zomba Central Hospital	84 (65.1)				
		Outside health facility	45 (34.8)				
**Weight and gestation**							
	**Birth weight (g; n=117)**			12 (9.3)		117 (90.7)	
		Mean (SD)	2616 (750)				
		Median (IQR)	2800 (2000-3200)				
		Range	600-4000				
		<2500 g (LBW^a^), n (%),	44 (37.6)				
		>2500 g (normal birth weight), n (%)	73 (62.4)				
	**Admission weight (g; n=129)**			0 (0)		129 (100)	
		Mean (SD)	2638 (841)				
		Median (IQR)	2750 (2030-2750)				
		Range	630-5320				
		<2500 g, n (%)	49 (38.0)				
		>2500 g, n (%)	80 (62.0)				
	**Gestation (weeks) by fundal height and LMP^b^ (n=129)**			0 (0)		129 (100)	
		Mean (SD)	36 (4)				
		Median (IQR)	37 (34-38)				
		Range	24-42				
		<37 weeks, n (%)	58 (45.0)				
		≤30 weeks, n (%)	9 (7.0)				
**Discharge diagnoses and outcome**							
	**Researcher-in-residence diagnosis (mutually exclusive^c^; n=129), n (%)**			0 (0)		129 (100)	
		Neonatal sepsis	44 (34.1)				
		Birth asphyxia (mild or moderate or severe)	30 (23.3)				
		LBW	13 (10.1)				
		Prematurity with RDS^d^	6 (4.7)				
		Pneumonia or bronchiolitis	6 (4.7)				
		Prematurity only	13 (10.1)				
		Congenital abnormality	6 (4.7)				
		Other	6 (4.7)				
		Well baby	4 (3.1)				
	**HCP^e^ discharge diagnoses (not mutually exclusive^f^; n=129), n (%)**			0 (0)		129 (100)	
		Neonatal sepsis	58 (45.0)				
		Birth asphyxia	28 (21.7)				
		Prematurity with RDS	20 (15.5)				
		Congenital anomaly	5 (3.9)				
		Other	42 (32.6)				
	**Outcome (n=121), n (%)**			8 (6.2)		121 (93.8)	
		Absconded	7 (5.8)				
		Discharged alive	102 (84.3)				
		Neonatal death	12 (9.9)				

^a^LBW: low birth weight.

^b^LMP: last menstrual period.

^c^Mutually exclusive: assumes one single diagnosis only.

^d^RDS: respiratory distress syndrome.

^e^HCP: health care professionals

^f^Not mutually exclusive: assumes on discharge some babies had more than one diagnosis, which may have contributed to their presentation.

**Table 3:** Findings at emergency triage and HIV status of babies.

Characteristics			Findings, n (%)	Missing, n (%)	Complete, n (%)
**Emergency triage, danger signs and vital signs**					
	**Baby crying triage (n=129), n (%)**			0 (0)	129 (100)
		Crying	42 (32.6)		
		Not crying	87 (67.4)		
	**ABCD^a^ triage (n=87), n (%)**			0 (0)	87 (100)
		Stable	76 (87.4)		
		Not stable	11 (12.6)		
	**Danger signs (n=129), n (%)**			0 (0)	129 (100)
		Grunting	18 (14.0)		
		Cold trunk	7 (5.4)		
		Prolonged capillary refill time	3 (2.3)		
		Cyanosis	1 (0.8)		
		Convulsions	1 (0.8)		
		Weak femoral pulses	0 (0)		
		None	101 (78.3)		
**Vital signs, mean (SD)**					
		Admission HR^b^ (bpm^c^; n=122)	141 (31)	7 (5.4)	122 (94.6)^d^
		Admission oxygen saturations in air (%; n=122)	90 (12)	7 (5.4)	122 (94.6)^d^
		Manual HR (bpm; n=7)	102 (20)	0 (0)	7 (100)
		Admission RR^e^ (bpm; n=129)	65 (18)	0 (0)	129 (100)
		Admission oxygen saturations in oxygen (%; n=24)	86 (20)	2 (7.7)	24 (92.3)
		Admission temperature (°C; n=129)	37.0 (1.6)	0 (0)	129 (100)
		Admission blood sugar (mmol/l; n=124)	4.9 (4.1)	5 (3.9)	124 (96.1)^d^
	**Abnormal vital signs (n=129), n (%)**			0 (0)	129 (100)
		Tachycardic (HR>160 bpm)	21 (16.3)		
		Hypoxic (oxygen saturations <90% in air)	26 (20.2)		
		Tachypneic (RR>60 bpm)	66 (51.2)		
		Hypothermic (temperature <36.5°C)	49 (38.0)		
		Hyperthermic (temperature >37.5°C)	52 (40.3)		
		Hypoglycemic (blood sugar <2.6 mmol/l)	9 (7.0)		
**HIV status**					
	**HIV status (n=129), n (%)**			0 (0)	129 (100)
		Exposed	25 (19.4)		
		Unexposed	101 (78.3)		
		Unknown	1 (0.8)		

^a^ABCD: airway, breathing, circulation, and disability.

^b^HR: heart rate.

^c^bpm: beats/breaths per minute for HR/RR, respectively.

^d^n=129.

^e^RR: respiratory rate.

**Table 4:** Cause of death and case fatality rates.

Cause of death^a^	Percentage deaths from total cases^b^, n (%)	Case fatality rate per 1000 cases	Percentage deaths from total deaths (n=12), n (%)
Prematurity with RDS^c^ (n=7)	3 (43)	428	3 (25)
Birth asphyxia (n=30)	7 (23)	233	7 (58)
Neonatal sepsis (n=44)	1 (2)	23	1 (8)
Congenital anomaly (n=6)	1 (17)	167	1 (8)
Total (N=129)	12 (9)	93	12 (100)

^a^Causes of death are mutually exclusive, that is, only 1 cause of death per neonate.

^b^Diagnosis refers to a researcher-in-residence’s discharge diagnosis, which was the same as the cause of death for all neonatal deaths.

^c^RDS: respiratory distress syndrome.

### Examination of the Newborn

A summary of findings on examination of the newborn is detailed in Multimedia Appendix 2. A total of 53 admissions (53/129, 41.1%) had signs of difficulty breathing or respiratory distress where chest in-drawings was the most commonly reported sign (36/129, 27.9%). On the basis of clinical judgement of the use of accessory muscles and prominence of chest retractions to aid breathing, 12 (12/129, 9.3%) were deemed to have severe work of breathing. Of HCPs, 56 (56/129, 43.4%) reported confidence in using a stethoscope; however, there was a lack of chest findings reported, and no heart murmurs were reported at all. Examination of the fontanelle was unavailable in the data because the confidential button had been pressed in error by the researcher-in-residence during the configuration of this field.

#### Place of Origin

In terms of place of birth, 93 (93/129, 72.1%) of the admissions were born in a hospital, 3 (3/129, 2.3%) were born at home, 27 (27/129, 20.9%) were born in a health center, and 4 (4/129, 3.1%) were born before arrival. Only 2 (2/129, 1.6%) were born with a traditional birth attendant despite a *ban* of traditional birth attendants by the government. Other facilities referred 40 patients (Multimedia Appendices 3 and 4).

#### Maternal and Antenatal History

**Figure 5:**
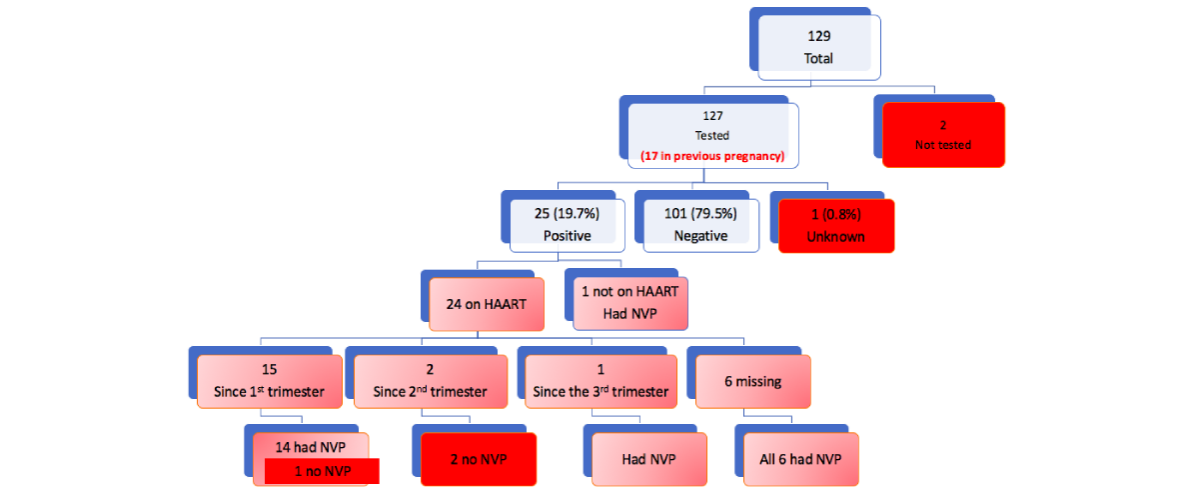
HIV status of mothers of babies admitted to neonatal unit using the NeoTree app. HAART: highly active antiretroviral therapy; NVP: nevirapine.

The mothers’ ages were poorly recorded as 62 (62/129, 48.1%) mothers did not know their exact date of birth, and it was not exported for analysis because it is potentially identifiable information. A summary of the maternal history captured by the app is shown in Multimedia Appendix 5. Attendance at antenatal care was generally poor (Multimedia Appendix 6), with 40 (40/129, 31.0%) attending 2 or fewer antenatal appointments. A minimum of 3 antenatal appointments is needed to receive all doses of the tetanus vaccine. Most mothers (127/129, 98.4%) had been tested for HIV, and 25 (25/127, 19.7%) of those tested had a positive result (Figure 5). Of the 3 HIV-exposed babies who did not receive nevirapine prophylaxis, 2 had mothers who delayed highly active antiretroviral therapy until the second trimester; hence, they were the most vulnerable to vertical transmission of HIV.

Syphilis status was much more poorly recorded in comparison with HIV status, with *unknown* status in 21 mothers (21/129, 16.3%). A total of 35 mothers (35/129, 27.1%) had definitely not had a syphilis test. Of the 73 (73/129, 56.6%) mothers who had been tested, 3 were positive, and all of their babies were treated with penicillin. Medical conditions in pregnancy included malaria (16/129, 12.4%), hypertension (2/129, 1.6%), other sexually transmitted diseases (2/129, 1.6%), and anemia (2/129, 1.6%). No maternal heart disease, diabetes, or thyroid disease was reported in NeoTree. Antenatal steroids were administered to 8 mothers (8/129, 6.2%).

#### Labor History

A summary of the data captured for labor history is shown in Multimedia Appendices 7 and 8. Maternal conditions reported in the labor field of the NeoTree app included significant vaginal bleeding seen in 4 mothers, but no other problems were reported.

### The Digital Process

#### Completeness of Data

Most fields in the app admission form were compulsory; hence, data for these fields were 100% complete with no missing data. For example, respiratory rate and temperature were recorded for all 129 babies in the sample (these were compulsory fields; Table 2). Only 5 admission fields were not compulsory and had less than 100% data completion rates: admission heart rate (HR; 122/129, 94.6%), admission oxygen saturation (122/129, 94.6%), saturations in oxygen (24/26, 92.3%), birth weight (117/129, 90.7%), and blood sugar (124/129, 96.1%). For the 7 missing admission HR and saturation readings (likely because of the lack of a pulse oximeter), the app directed the HCP to take and record a manual HR; hence, some form of HR (electronic or manual) was recorded for every baby. There were 8 missing outcomes at discharge because of a lack of clear outcome documentation in the patient record.

#### Time Taken

The mean time taken to complete an admission using the NeoTree app was 37 min (range 18-59 min), excluding 1 outlier (n=1). Anecdotal reports from HCPs suggest that longer sessions may have been interrupted by urgent tasks. Approximately one-fifth (22%) of NeoTree admissions were supervised.

### Emerging Patterns and Corresponding Modifiable Factors Discussed: Stage 4 of the Audit Cycle

The audit meeting took just over 1 hour, after which HCPs could return to their daily duties; 4 factors were discussed. First, the high rate of hypothermia on admission (Table 3) and thermoregulation of babies by drying and wrapping was identified as a modifiable factor for improvement and future reauditing. Hypothermia is also an example of a factor that could be highlighted in the anticipated next phase of NeoTree development, that is, feedback data dashboards linked to the NeoTree data. Second, inadequate reporting of antenatal syphilis testing was discussed, and it was suggested that this could be fed back to health centers via the District Health Officer. Furthermore, the timing and completeness of penicillin treatment were requested to be added to the NeoTree form. Third, difficulties in knowing mothers’ age were also highlighted as important because younger mothers typically experience more premature deliveries and more complications of pregnancy. A request was made for the app to calculate this in the future. Finally, because of the lack of reported problems in labor other than bleeding, it was deliberated that this question may have been answered poorly and that midwives and newborn HCPs should be shown where to find this information.

## Discussion

### Principal Findings

This paper presents a novel approach to capturing documentation in an inpatient setting that could signal a start to inpatient computerization. To our knowledge, this is the first digital outcome audit of neonatal admissions to a low-resource newborn facility. Our digital outcome audit achieved 70% coverage of admissions during a phased implementation approach where HCPs collected the data themselves on a novel app, NeoTree [10]. The overall CFR for newborns admitted on NeoTree was 92 per 1000. The most common diagnoses were sepsis, prematurity, and birth asphyxia for which the CFRs were 34, 250, and 250 per 1000, respectively. The completeness of data was high or 100% for much of the data set, exemplifying how the digital method significantly improves the quality of data in terms of completeness. In comparison, other studies have commented on how >50% of the charts had missing documentation [12]. Our 1-month audit has completed steps 1 to 4 of the audit cycle (Figure 1) [5] and has the potential for reaudit, evaluation, and refinement of recommendations and hence the completion of the whole audit cycle.

### Discussion of Findings in Context

In a previous study at the ZCH nursery, demographic data were collected over a 2-month period using the MOH paper admission form. The mortality rate was 160 per 1000, significantly higher than that of our digital outcome audit [17]. Therefore, our digital outcome audit could have underestimated CFRs because of systematically missing data on babies who died. As HCPs were required to complete a paper form in addition to NeoTree, they may not have filled out a NeoTree admission form for babies that died soon after birth. If the NeoTree app completely replaced the paper option and HCPs were trained to clerk all babies, including those arriving moribund, this could cease to be a problem.

Another previous paper-based death audit in KCH hospital, Malawi, audited pediatric patients, with ages ranging from 1 day to 16.5 years, rather than newborns, and showed mortality rates ranging from 22 to 44 per 1000 [12]. The lower mortality rates most likely reflect the older age range, but may also be because of the retrospective nature of their study and missing data. The authors reported that >50% of the charts had missing documentation [12]. The prospective nature of our study, the presence of a researcher onsite overseeing data collection, and the use of a digital method may have aided in improving the completeness of data in our study.

Field validation and compulsory fields within the app may have also contributed to the completeness of data. Saturations in oxygen, for example, were not a compulsory field and were recorded in 24 of 26 babies. This may reflect the power of compulsory fields but also a lack of time to wait for a second saturation reading once oxygen had been applied. Indeed, the adult pulse oximeters available took time to pick up a reading, particularly in smaller premature infants. Local protocols (care of the infant newborn) [16] specify how to measure oxygen saturation and that it should be taken in air and oxygen as part of the assessment for starting continuous positive airway pressure, but these were newly implemented at the time of the study. For optional fields, lack of available information (eg, birth weight of older infants born in other facilities) and equipment (eg, test strips for the blood sugar monitor) may have contributed to incomplete data, and these fields were intentionally configured as optional for these reasons. For other fields (eg, syphilis status), an *unknown* option was added to allow progress through the app, where data were unavailable. A researcher collected the outcome data in our study by reading the documentation of HCPs (which was only 94% complete); hence, in the future, the recording of outcomes by the HCPs themselves in real time might improve the completeness of outcome data. Although the completeness of data was generally high, there is always the possibility of false data being entered. Since we did not include any quality assurance in our study, we can only assume that HCPs were entering correct data.

### Discussion of Key Fields Within the NeoTree App

The percentage of LBW at admission (<2.5 kg) may be a useful indicator for the procurement of feeding cups and nasogastric tubes and the provision of kangaroo mother care beds. These data could also potentially influence the maternal and obstetric department and potentially government policies to tackle the nutrition of Malawian mothers and their babies.

It is important to note that LBW or small for gestational age is not the same as prematurity; hence, a maturity score was included within NeoTree. The difference in maturity scores and estimated gestation exposed the inaccuracy of fundal height and length of pregnancy, suggesting a significant underestimation of gestation using these methods. Feedback that the maturity score was time-consuming and required additional training prompted its removal from NeoTree halfway through the study.

Our results from the subjective assessment of the severity of work of breathing (WOB) suggest that nasal flare and chest in-drawings were not considered *severe* WOB. Head nodding, grunting, and tracheal tug made up for 9.3% of severe WOB. This could be further analyzed in the next phase to improve the understanding of the training needs of HCPs in assessing respiratory distress and potentially develop a scoring system in the future.

Regarding the examination of the newborn, the lack of chest findings reported and the complete lack of heart murmurs auscultated suggest that related fields may not be appropriate for nursing cadres, but their relevance for doctors could be examined in the future. The flexible nature of the NeoTree app means that these fields can be optional. For head circumference and birth weight, the app could ideally plot these automatically on a growth chart. However, it may represent a training challenge for HCPs to interpret these. Nevertheless, this is certainly a consideration for future iterations.

### Limitations

The coverage of admissions was only 70.2% during a phased implementation; hence, this may not be a representative sample. The paper charts for the remaining 29.8% could have been checked to improve coverage of key indicators such as age and sex; however, this was not considered a reliable alternative because of missing charts and missing documentation [12]. As this was part of a proof-of-concept study, the digital form had to be completed in addition to the paper form, adding time and workload to already pressured staff. The researcher-in-residence was present throughout the study, which may have enhanced uptake. There were difficulties completing antenatal fields, particularly when a guardian accompanied the infant to the nursery while the mother was still recovering in the labor ward. As the study progressed, midwives started to bring the mother’s labor ward notes, in addition to her hand-held record, with new admissions from the labor ward, but the problem persisted for out-born babies. Hence, the option *unknown* was added to many of the drop-down menus to preserve practical feasibility.

A major problem with our digital outcome audit is that the proposed system only collected data at the point of admission and discharge. What occurred during the crucial period between admission and death was not recorded, and therefore modifiable factors contributing to deaths and reciprocal solutions could not be identified. In turn, because of time constraints, steps 5 and 6 of the audit cycle could not be executed, leaving the audit loop unclosed. However, we have identified a considerable number of modifiable factors from patterns in aggregate data; hence, with the right resources and staff available, we could potentially close the audit loop.

### Future Steps

To allow the scrutiny of individual causes of death, a free text field will be added to the *NeoDischarge* form for the reviewing clinician to record (in a nonblame anonymous fashion) any possible modifiable factors that might have prevented that death. Copies of these *death summaries* could potentially be printed and collated for review in monthly death audits, which would significantly increase the efficiency of these meetings and provide valuable contemporary insights into how and why an individual newborn died. Other next steps include using NeoTree where it completely replaces the paper form, or where no paper form exists in the first place, without the presence of the researcher on site. A study where clinicians or doctors use the app in another low-resource country would also be recommended.

### Conclusions

Using an mHealth app, NeoTree, a digital outcome audit was successfully carried out by health care workers at a neonatal unit of a district hospital in Malawi with high completeness of data. These results were discussed at a local audit meeting and demonstrated that data collected digitally could stimulate quality improvement initiatives, such as improving the thermoregulation of babies. Limitations are noted in this study, with only 70% coverage of all admissions. Overall, this study illustrates how a digital audit using an app can improve documentation and richness of clinical data to help support the delivery and configuration of local services. This study demonstrates huge potential for the use of a daily electronic record in low-resource settings, and these findings can inform the next stage of development for the NeoTree app, in particular, for guiding the development of linked data dashboards.

## Appendix

Multimedia Appendix 1 Maturity scores compared to fundus height and length of pregnancy measured.

Multimedia Appendix 2 Findings on newborn examination.

Multimedia Appendix 3 Place of origin.

Multimedia Appendix 4 Map of referral centres showing number of referrals from outlying referral centres.

Multimedia Appendix 5 Maternal history.

Multimedia Appendix 6 Summary of antenatal care visits and medications/vaccinations received.

Multimedia Appendix 7 Summary of characteristics of labour as captured by the NeoTree app.

Multimedia Appendix 8 Reported reasoning for either elective or emergency C-section.

## Abbreviations

CFR: case fatality rate
HCP: health care professional
HR: heart rate
KCH: Kamuzu Central Hospital
LBW: low birth weight
mHealth: mobile health
MOH: Malawian Ministry of Health
NHS: National Health Service
NIHR: National Institute for Health Research
NMR: neonatal mortality rate
NND: neonatal death
RDS: respiratory distress syndrome
WOB: work of breathing
ZCH: Zomba Central Hospital
