# Supplementary material for: Admissions to a Low-Resource Neonatal Unit in Malawi Using a Mobile App: Digital Perinatal Outcome Audit
Source: JMIR Mhealth Uhealth. 2020 Oct 21;8(10):e16485. doi: 10.2196/16485 (PMC7641784; doi:10.2196/16485)
Supplement: Multimedia Appendix 1 [file mhealth_v8i10e16485_app1.pdf]

Patient number	Weeks gestation using Fundal height/ Length of Pregnancy	Weeks gestation using Maturity Score from COIN guidelines[a]
**1**	40	43
**2**	36	42
**3**	37	41
**4**	35	40
**5**	32	39
**6**	28	37
**7**	32	37
**8**	34	36
**9**	36	36
**10**	36	36
**11**	34	35
**12**	34	35
**13**	28	35
**14**	36	35
**15**	30	34
**16**	34	34
**17**	28	31
**18**	32	31
**19**	24	31
**20**	32	29
**21**	24	28
**22**	26	28
**23**	33	27
**24**	32	20
**25**	30	31

[a] COIN = Care of the Infant Newborn neonatal guidelines for Malawi [13]
