# Supplementary material for: Admissions to a Low-Resource Neonatal Unit in Malawi Using a Mobile App: Digital Perinatal Outcome Audit
Source: JMIR Mhealth Uhealth. 2020 Oct 21;8(10):e16485. doi: 10.2196/16485 (PMC7641784; doi:10.2196/16485)
Supplement: Multimedia Appendix 2 [file mhealth_v8i10e16485_app2.pdf]

**Respiratory / Cardiovascular**		**Neurology**		**Gastro**	
Examination	Findings	Examination	Findings	Examination	Findings
**Signs of respiratory distress[a] (N=129) n(%)**		**Head circumference (cm)**		**Abdomen (N=129) n(%)**	
Nasal Flare	25 (19.4%)	Mean	34.5	Distension	8 (6.2%)
Chest In-drawings	36 (27.9%)	Range	22 - 45	Defect	1 (0.8%)
Head Nodding	3 (2.3%)	**Head shape (N=129) n(%)**		Normal	119 (92.2%)
Grunting	5 (3.9%)	Normal	120 (93.0%)	Missing	1 (0.8%)
Tracheal tug	3 (2.3%)	Molding	5 (3.9%)	**Umbilicus (N=129) n(%)**	
**Severity of WOB (N=129) n(%)**		Head swelling	2 (1.6%)	Infected	3 (2.3%)
Mild	24(18.6%)	Hydrocephalus	1 (0.8%)	Meconium stained	2 (1.6%)
Mod	17(13.2%)	Missing	1 (0.8%)	Abnormal	1 (0.8%)
Severe	12(9.3%)	**Suck reflex (N=129) n(%)**		Bloody	1 (0.8%)
**Confident using a stethoscope (N=129) n(%)**		Present but weak	16 (12.4%)	Normal	121 (93.8%)
Yes	56 (43.4%)	Absent	15 (11.6%)	Missing	1 (0.8%)
No	73 (56.6%)	Strong	97 (75.2%)	**Anus (N=129) n(%)**	
**Chest Auscultation (N=129) n(%)**		Missing	1 (0.8%)	Abnormal	1 (0.8%)
Clear	54 (41.9%)	**Tone (N=129) n(%)**		Imperforate	1 (0.8%)
Unequal air entry	3 (2.3%)	Low (hypotonic)	12 (9.3%)	Patent	126 (97.7%)
Unilateral crackles	0 (0%)	High (hypertonic)	1 (0.8%)	Missing	1 (0.8%)
Unilateral wheeze	0 (0%)	Normal	115 (89.1%)	**Genitalia (N=129) n(%)**	
Bilateral crackles	4 (3.1%)	Missing	1 (0.8%)	Abnormal male	1 (0.8%)
Stridor	2 (1.6%)	**Spine (N=129) n(%)**		Abnormal female	1 (0.8%)
Missing	66 (51.2%)	Neural tube defect	1 (0.8%)	Male	77 (60.0%)
**Heart sounds (N=129) n(%)**		Other abnormality	2 (1.6%)	Female	49 (38.0%)
Normal	128 (99.2%)	Normal	125 (96.9%)	Missing	1 (0.8%)
Murmurs	0 (0%)	Missing	1 (0.8%)	**Musculoskeletal (N=129) n(%)**	
Missing	1 (0.8%)	**Activity (N=129) n(%)**		Rash	2 (1.6%)
**Signs of dehydration (N=129) n(%)**		Lethargic	15 (11.6%)	Talipes	2 (1.6%)
Prolonged skin pinch	4 (3.1%)	Irritable	4 (3.1%)		
Dry mucous membranes	2 (1.6%)	Convulsing	1 (0.8%)		
No signs of dehydration	100 (77.5%)	Alert	108 (83.7%)		
Missing	23 (17.8%)	Missing	1 (0.8%)		

[a]N.B these results are not mutually exclusive because in some cases there was more than one sign of respiratory distress present
