# Supplementary material for: Admissions to a Low-Resource Neonatal Unit in Malawi Using a Mobile App: Digital Perinatal Outcome Audit
Source: JMIR Mhealth Uhealth. 2020 Oct 21;8(10):e16485. doi: 10.2196/16485 (PMC7641784; doi:10.2196/16485)
Supplement: Multimedia Appendix 3 [file mhealth_v8i10e16485_app3.pdf]

Place of origin		n (%)
**Place of birth (N=129)**		
	Hospital	93 (72.1)
	Health Centre	27 (20.9)
	Born Before Arrival (on the way to a health care facility)	4 (3.1)
	Home	3 (2.3)
	Traditional birth attendant (TBA)	2 (1.6)
**Referral facility (N = 40)[a]**		
	Machinga District Hospital	4 (10)
	Matawale HC	4 (10)
	Mangochi District Hospital	3 (8)
	M'mambo HC	3 (8)
	Ngwelero HC	3 (8)
	Bimbi HC	2 (5)
	Domasi Rural Hospital	2 (5)
	Magomero HC	2 (5)
	Makwapala HC	2 (5)
	Matiya HC	2 (5)
	Other facilities	10 (25)

[a] There were 20 different referral facilities in total.

HC = Health Centre
