# Supplementary figures and images for: Admissions to a Low-Resource Neonatal Unit in Malawi Using a Mobile App: Digital Perinatal Outcome Audit
Source: JMIR Mhealth Uhealth. 2020 Oct 21;8(10):e16485. doi: 10.2196/16485 (PMC7641784; doi:10.2196/16485)

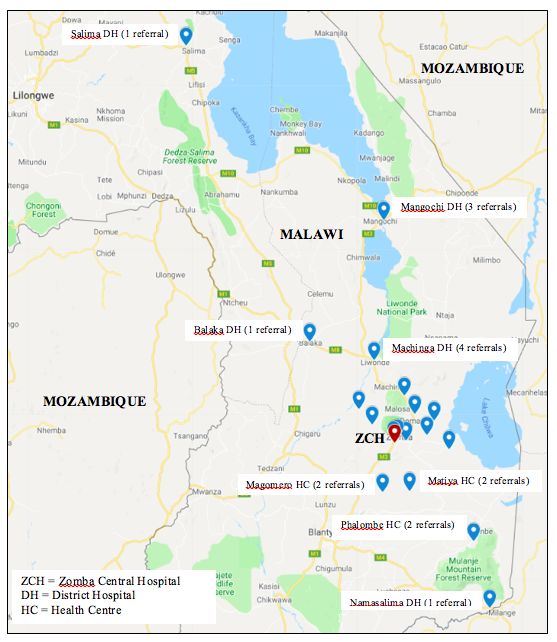

Supplement: Multimedia Appendix 4 [file mhealth_v8i10e16485_app4.png]

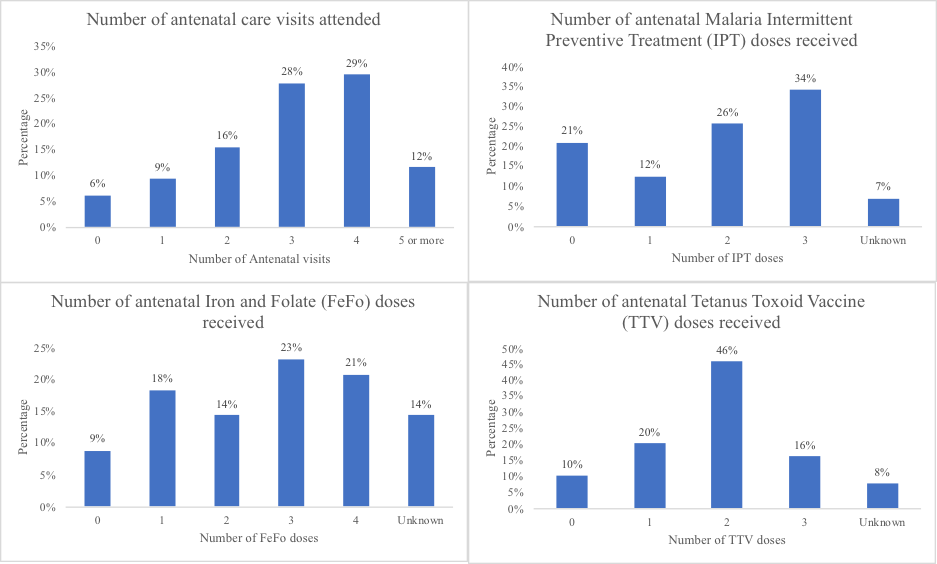

Supplement: Multimedia Appendix 6 [file mhealth_v8i10e16485_app6.png]
