# Supplementary material for: Admissions to a Low-Resource Neonatal Unit in Malawi Using a Mobile App: Digital Perinatal Outcome Audit
Source: JMIR Mhealth Uhealth. 2020 Oct 21;8(10):e16485. doi: 10.2196/16485 (PMC7641784; doi:10.2196/16485)
Supplement: Multimedia Appendix 5 [file mhealth_v8i10e16485_app5.pdf]

**Maternal Characteristics**		**n (%)**
**Marital status (N=129)**		
	Married	115 (89.1)
	Single	11 (8.5)
	Divorced	3 (2.3)
**Ethnicity (N=129)**		
	Malawian	129 (100.0)
**Tribe**[a] (N=129)		
	Lomwe	39 (30.2)
	Yao	38 (29.5)
	Chewa	26 (20.2)
	Other	26 (20.2)
**Religion**[b] (N=129)		
	Roman Catholic	29 (22.5)
	Muslim	23 (17.8)
	Church of Christ	12 (9.3)
	Church of Central Africa (CCAP)	10 (7.8)
	Seventh Day Adventist	10 (7.8)
	Other	45 (34.9)

[a] There were 11 different tribes in total

[b]There were 36 different religions in total
