# Supplementary material for: Admissions to a Low-Resource Neonatal Unit in Malawi Using a Mobile App: Digital Perinatal Outcome Audit
Source: JMIR Mhealth Uhealth. 2020 Oct 21;8(10):e16485. doi: 10.2196/16485 (PMC7641784; doi:10.2196/16485)
Supplement: Multimedia Appendix 7 [file mhealth_v8i10e16485_app7.pdf]

Labour history		All (N = 129)
**Type of Labour, n(%)**		
	Spontaneous	121 (93.8)
	Induced	8 (6.2)
**Duration of Labour (Hours), Mean(Range)**		12.3 (0-96)
**Rupture of membranes, n(%)**		
	Yes	90 (69.7)
	No	39 (30.2)
**Length of rupture of membranes (hours), Mean(Median)**		13 (4)
**Risk Factors for Sepsis, n(%)**		
	Any risk factor present	47 (36.4)
	Prematurity <37 weeks	26 (20.2)
	Prolonged rupture of membranes (PROM)	8 (6.2)
	Prolonged second stage	6 (4.7)
	Born before arrival (BBA)	5 (3.9)
	Maternal fever	3 (2.3)
	Offensive liquor	1 (0.8)
**Type of Delivery, n(%)**		
	Spontaneous vaginal delivery (SVD)	96 (74.4)
	Emergency caesarean section (EMCS)	19 (14.7)
	Elective caesarean section (ELCS)	12 (9.3)
	Vacuum Extraction	1 (0.8)
	Forceps[a]	0 (0.0)
**Features at Delivery, n(%)**		
	Meconium present	17 (13.2)
	Cried straight after birth	89 (69.0)
	5 minute Apgar score <6	7 (5.4)
	Required BVM[b] at birth	16 (12.4)
	Required oxygen or more for resuscitation	20 (15.3)
**Length of resuscitation (mins), Mean(Range)**		16 (1-60)

[a]This is consistent with the ban on forceps deliveries in Malawi due to associated high trauma rate.

[b]BVM = Bag-valve-mask ventilation
