# Supplementary material for: Admissions to a Low-Resource Neonatal Unit in Malawi Using a Mobile App: Digital Perinatal Outcome Audit
Source: JMIR Mhealth Uhealth. 2020 Oct 21;8(10):e16485. doi: 10.2196/16485 (PMC7641784; doi:10.2196/16485)
Supplement: Multimedia Appendix 8 [file mhealth_v8i10e16485_app8.pdf]

**Reasons for elective caesarean-section (n =12)**	**Number, n(%)**	**Reasons for emergency caesarean-section (n = 19)**	**Number, n(%)**
Placental calcification	2 (16)	Foetal distress	4 (21)
Previous scar	2 (16)	Prolonged labour	4 (21)
Big fundus	2 (16)	Big fundus	4 (21)
Multiple gestation	1 (8)	Antepartum haemorrhage	3(16)
Malpresentation	1 (8)	Malpresentation	2(10)
Cephalopelvic disproportion	1 (8)	Cephalopelvic disproportion	1(5)
		Failure to progress	1(5)
		Previous scar	1(5)
		Syphilis	1(5)

N.B these results are not mutually exclusive because in some cases there was more than one reason for the caesarean section.
